# Effect of extremely low frequency electromagnetic field exposure on sleep quality in high voltage substations

**DOI:** 10.1186/1735-2746-9-15

**Published:** 2012-11-30

**Authors:** Tayebeh Barsam, Mohammad Reza Monazzam, Ali Akbar Haghdoost, Mohammad Reza Ghotbi, Somayeh Farhang Dehghan

**Affiliations:** 1 Department of Occupational Health, School of Public Health, Kerman University of Medical Sciences, Kerman, Iran; 2 Department of Occupational Health, School of Public Health and Center for Air Pollution Research (CAPR), Institute for Environmental Research (IER), Tehran University of Medical Sciences, Tehran, Iran; 3 Department of Epidemiology, School of Public Health, Kerman University of Medical Sciences, Kerman, Iran; 4 Department of Occupational Health, School of Public Health, Tehran University of Medical Sciences, Tehran, Iran

**Keywords:** Electric and magnetic field, Extremely low-frequency, Sleep quality, Pittsburgh sleep quality index

## Abstract

This study aims to investigate the effect of extremely low frequency electromagnetic fields exposure on sleep quality in high voltage substations (132, 230 and 400 KV) in Kerman city and the suburbs. For this purpose, the electric field intensity and magnetic flux density were measured in different parts of substations, and then the occupational exposure was estimated by averaging electric field intensity and magnetic flux density in a shift work. The cases comprised 67 workers who had been exposed to electromagnetic fields in age range of 24–57 and the controls were 110 persons the age ranged 24–50 years. Sleep quality of both groups was evaluated by the Pittsburgh Sleep Quality Index questionnaire (PSQI). Finally, these data were subjected to statistical analysis. The results indicated that 90.5% of cases and 85.3% of controls had the poor quality sleep according to PSQI (P-value=0.615). Total sleep quality score mean for the case and control groups were 10.22 ± 3.4 and 9.74 ± 3.62 (P-value=0.415) ,respectively. Meantime to fall asleep for cases(35.68 ± 26.25 min) was significantly higher than for controls (28.89 ± 20.18 min) (P-value=0.002). Cases had average sleep duration of 5.49 ± 1.31 hours, which was lower ascompared with control subjects (5.90 ± 1.67hours). Although there was a higher percentage for the case group with poor sleep quality than the control group, but no statistically significant difference was observed.

## Introduction

Electromagnetic fields (EMFs) are produced by production, transmission, and use of electrical devices such as power lines, electrical wiring, transformers and electrical equipments, so each person in the society can be exposed to them [1]. Extremely low frequency (ELF) fields includes alternating current (AC) fields and other electromagnetic, and non-ionizing radiation from 3Hz to 300Hz [2]. Electric power substations, transmission lines, distribution lines, industrial devices as well as electric appliances are some of the commonly known sources of ELF magnetic fields in the environment. Since many substations are surrounded by residential or commercial areas, people living nearthemand alsopersonnel working in the substations may be exposed to high ELF magnetic fields [3]. As a result, it is necessary to assess personal exposure to ELF magnetic fields and the effects of them [4].

Recent studies have been done mostly on the health effects of exposure to non-ionizing low frequency radiation. It has been shown that different types of animals, and humansare sensitive even to minor exposure to electromagnetic fields [3,5]. Public concerns have been raised about possible effects of exposure to electromagnetic fields on human health and [2]. EMF can lead to rapid aging and have a significant impact on metabolic systems including elevated blood glucose levels, elevation in lipid levels, increased neuroregulatory disturbances, decreased testosterone levels in males and impacts on the Central nervous system (CNS), cardiovascular, and immune systems amongst other issues. EMFs have an effect on raised blood pressure, pulse rate and affecting other dynamics of cardiovascular function [6,7]. Recently, several studies reported that extremely low-frequency magnetic field exposure might disrupt normal sleep [2]. Asanova and Rakov were the first to note that occupational exposure to power frequency electric and magnetic fields might have a detrimental influence on night sleep. Akorstedt observed that magnetic field exposure was associated with a significant reduction in the duration of slow wave sleep (sleep stages III and IV) [8]. In 2009, Sharifi reported the prolonged exposure to magnetic fields of 230 kV high voltage substations can cause to increase the psychiatric disorders like sleep disturbance, despite that the exposure level was lower than occupational permissible limits (ICNIRP) [9]. Yousefi observed magnetic field exposure can exacerbate some psychological problems. He considered 103 workers in Tehran high voltage substations. Thesymptoms of depression, paranoia, intellectual and practical obsession, interpersonal sensitivity, anxiety, hostility, phobia and psychosis were observed based on questionnaire results [10].

Sleep, a complex biological process controlled by the central nervous system, is necessary for proper cognitive, metabolic, and immune function [8].We spend a third of our life asleep because sleep is vital to everyone**.** Studies have shown a direct link between the amount and quality of sleep and our abilities of the next day and also disruption to sleep, especially ongoing problems, affects our quality of life and can have a bad effect on our health and general well-being [11]. Night sleep is tonic and will help to daily good performance [12]. Sleep allows our body and mind to rest [13]. Graham and Cook reported that an intermittent, but not a continuous, magnetic field exposure may disrupt sleep. Electro-biological research of sleep showed that EMF at night has more severe effects on health in comparision to EMF radiation in day time [2]. Poor sleep quality has been associated with increased tension, irritability, depression, confusion and generally lower life satisfaction [14]. Poor sleep quality reduces longevity from 80 years to 65–70 years [15]. Since poor sleep quality is associated with numerous health problems (gastrointestinal disorders, depression, exacerbation of chronic disease) and extremely low frequencies are in the frequency ranges of brain waves and also they have more impact on employee performance, we the sleep quality in the workers of high voltage substations in the city of Kerman and its suburbs was assessed. Kerman is the largest and most developed city in the Kerman Province and also the major city in South-East of Iran.In addition, workers in high voltage electricity substations are exposed to ELFMF more than other people [2].

## Materials and methods

The domain of the activity taken into account was 132, 230 and 400 KV substations in Kerman city and its suburbs (Baghyn, Kerman, Shahab, Tavakol Abad, Zangy Abad, Rhine, Mahan, Syrach and Nirogah). In order to assess the effect of ELFMF exposure on sleep quality in these substations, a case–control study was carried out. The cases comprised 67 workers who had been exposed to electromagnetic fields in the age range 24–57 years and the controls were 110 people in the age range of 24–50 years. In 132 KV substations, 41 cases of were chosen, 20 person in 230 KV substations; and the rest were in the 400 KV substations. all participants were male. Workers of cement factory, Barez tire and Bahonar copper industries constituted the control group who worked in same geographic space of high voltage substations but were far from them. Since high voltage substation operators were the shift workers; control group was selected from shift workers too. Another reason for choosing shift workers was to moderate the effect of shift work on sleep quality in both groups, because the change in circadian rhythm is one of the main causes of sleep disorder in shift workers [16-19]. Smokers, people with cardiovascular, pulmonary, diabetes and other diseases were excluded by the demographic questionnaire to prevent the interference in sleep. 15 high voltage substations, three 230 KV substations and twelve 132KV substations, and one ultra-high voltage substation (400 kV) were inspected. Then, the stations were determined according to the area of each substation, the amount of time that an operator spent in different parts and the distance from equipment. Finally, number of samples was determined in each substation and electric field intensity and magnetic-flux density were measured based on standard IEEESTD 644 – 1994 [20].

HI-3604 ELF survey meter is used to measure the magnetic flux density and electric field intensity. This device is designed for frequency range of 30–2000 Hz. Its sensitivity is 0.2 mm to 20 Gauss and 1 V/m to 200 kV/m. The electric field strength under power lines was measured at a height of 1 m above ground level. The probe was oriented to read the vertical E-field, because this quantity is often used to characterize induction effects in objects close to ground level. The distance between the electric field strength meter and operator was at least 2.5 m. The distance between the meter and nonpermanent objects was at least three times the height of the object in order to measure the unperturbed field value. The distance between the meter and permanent objects was ~1 m or more to ensure sufficient measurement accuracy of the ambient perturbed field. A remote control system was used to read the electric field values [21,22]. The consumption load of each substation was recorded during measurements. Since weather conditions can affect the results, all measurements were done between the hours of 4 to 9 pm in summer and in the sunny days.

Occupational exposure (time-weighted average ;TWA) to ELF electromagnetic fields in a shift work was calculated by following equation:

$$BC=\frac{B\left(t\right)i\times hi}{h}$$

Where:

BC = Occupational exposure

B(t)i= The mean magnetic flux density in different parts of each substation

hi= The mean time spent by operator for a specific duty in different parts of substations(hour)

h=Time shift (24 hours and 12 hours) [23-25].

Finally the results were compared with standard ICNRIP [26,27].

Data collection tool for investigating the operator's sleep quality was the Pittsburgh Sleep Quality Index questionnaire (PSQI). Validity of this questionnaire using Cronbach's alpha coefficient is 0.83 [14,28]. It checks the patient attitude about sleep quality in a one-month period. The seven component-scores are then summed to yield a global PSQI score, which has a range of 0–21; higher scores indicate worse sleep quality. These components are: subjective sleep quality, sleep latency, sleep duration, habitual sleep efficiency, sleep disturbances, use of sleeping medications, and daytime dysfunction. Previous studies have shown a remarkable agreement between the results of PSQI and sleep laboratory studies using polysomnography (PSG). Each questionnaire scale takes score of zero to three. Score of zero, one, two and three respectively in each scale represents the normal situation, mild, moderate and severe problem.Total score five and less means having a good sleep quality and score six and higher means having a poor sleep quality [14,29-33].

Before handing out the questionnaire the researcher explained the aims of the research and the method of questionnaire completion.Case and control subjects completed the questionnaire on three consecutive days. Totally 531 questionnaires were collected: 330 questionnaires for control group and 201 questionnaires from case group. In addition, the demographic questionnaire was completed by both groups. Finally the collected data were analyzed using descriptive statistical methods, independent-samples t-test, repeated measures (ANOVA), Chi - square and Pearson correlation.

## Results

Total number of measurement points was 2583 of which 1343 were related to the magnetic fields and 1240 to electric fields. We tried to determine whether the field levels exceeded or not the reference levels of the ICNIRP guidelines. Result showed that the occupational exposure to an intensity of electric field mean (E) and magnetic flux density mean (B) was below the recommended level (E> 10 kV/m and B >5000 mG) (Table 1). According to demographic data, mean age and body mass index (BMI) in the case group were 34.66 ± 9.18 yr, 24.45 ± 3.73 kg/m^2^ respectively and in the control group were 34.46 ± 6.13 yr, 25.1 ± 4.57 kg/m^2^. Age and BMI were not significantly different between the two groups (P-value=0.348, and 0.257, respectively). According to statistical analysis, the difference of marital status between the two groups was not statistically significant (P-value=0.132), but there was a statistically significant difference between the education level of case and control groups (P-value<0.0001).

**Table 1:** Mean (standard deviation) of intensity electric field (kV/m) and magnetic flux density (mG) received by operators in a shift work

**Substation**	**Number of substations**	**Number of operators**	**Number of measurement points**	**Mean (SD) intensity electric field**	**Mean (SD) magnetic flux density**
132 KV	12	41	1821	0.266(0.312)	2.32(1.32)
230 KV	3	20	607	0.681(0.508)	7.49(3.61)
400 KV	1	6	155	0.838(0)	7.51(0)

According to the results presented in Table 2, the average score of the PSQI was in cases 10.22 ± 3.4 and controls 9.74 ± 3.62 for case and content groups, respectively and no significant differences were seen between groups (P-value =0.415). Also, PSQI score was not significantly different between first, second and third times of the two groups (P-value =0.385, 0.672, 0.296, respectively). As mentioned above education level did not indicate a statistically significant difference between the two groups, so the relationship between education and quality of sleep in two groups was evaluated by repeated measures (ANOVA test). The result of this analysis show thatthere was no significant difference between education and quality of sleep (P-value=0.264). Percentage of good sleep quality in cases group was 9.5% and percentage of poor sleep quality was 90.5%. These values in control group were 11.4%, and 85.3% respectively. Number of mean total PSQI scorein terms of being good and poor were not significantly differenent between groups (P-value =0.615).

**Table 2:** Mean (SD) PSQI score of case and control groups in three times (Independent-samples t- test)

**Sleep Quality Total score**	**Group**	**Mean**	**SD**	**P-value**
First	Case	10.22	3.4	0.385
	Control	9.75	3.62	
Second	Case	10.01	3.35	0.672
	Control	9.75	3.65	
Third	Case	10.5	3.37	0.296
	Control	9.55	3.82	
Mean PSQI total	Case	10.22	3.4	0.415
	Control	9.74	3.62	

**Figure 1:**
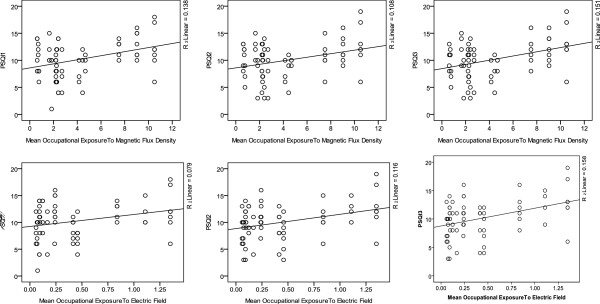
Occupational exposure to mean electric field intensity and magnetic flux density and its relationship with sleep quality score in three times.

Numbers of good or poor sleep quality did not show significant difference between first, second and third timesin the two groups (P-value =0.35, 0.78 and 0.78, respectively) (Table 3). Table 4 shows there was a linear relationship betweenoccupational exposure to mean intensity of electric field and magnetic flux density and their sleep quality score in every three times in different substations . This relationship became statistically significant. Exposure and sleep quality score are shown as scatter plot (Figure 1). Mean sleep quality scores in 132, 230 and 400 KVsubstations were 9.22 ± 3.14, 11.55 ± 3.4 and 11.77 ± 2.62, respectively (P-value<0.0001).

**Table 3:** Number and percentage of good and poor sleep quality in cases and controls (Chi- square test)

**Sleep quality total score**		**Group**		**P-value**
		**Control number (%)**	**Case number (%)**	
First	Good	13 (10.5)	5 (7.5)	0.352
	Poor	97 (78.2)	62 (92.5)	
Second	Good	13 (10.5)	7 (10.4)	0.780
	Poor	97 (78.2)	60 (89.6)	
Third	Good	13 (10.5)	7 (35)	0.780
	Poor	97 (78.2)	60 (89.6)	
Mean PSQI total	Good	39 (11.4)	19 (9.5)	0.615
	Poor	291 (85.3)	182 (90.5)	

**Table 4:** Correlation coefficient between average electric field intensity and magnetic flux density and PSQI total score (Pearson correlation test)

**Case group**	**Sleep quality score in first time**	**Sleep quality score in second time**	**Sleep quality score in third time**
Average magnetic flux density	0.371	0.328	0.388
P- value	0.002	0.007	0.001
Average electric field intensity	0.281	0.341	0.397
P- value	0.021	0.005	0.001

According to the results presented in Table 5, statistically significant finding was not found in PSQI Component Scoresbetween groups (P-value =0.156). The time interval mean going to bed for falling asleep , and duration of actual sleep in both groups showed statistically significant difference respectively, (P-value =0.002 and 0.002).

**Table 5:** PSQI component scores (Mean & SD) in case and control group (independent-samples t- test)

**Scale**	**Groups**	**First time**		**Second time**		**Third time**		**Total**	
		**Mean(SD)**	**P-value**	**Mean(SD)**	**P-value**	**Mean(SD)**	**P- value**	**Mean(SD)**	**P-value**
Subjective sleep quality	Case	1.33(0.76)	0.678	1.37(0.795)	0.639	1.40(0.779)	0.213	1.36(0.783)	0.156
	Control	1.28(0.692)		1.32(0.729)		1.25(0.747)		1.28(0.721)	
Sleep latency	Case	1.54(0.974)	0.440	1.48(1.005)	0.517	1.51(1.006)	0.509	1.54(1.01)	
	Control	1.43(0.883)		1.38(0.919)		1.41(0.932)		1.40(0.908)	
Sleep duration	Case	1.75(1.005)	0.430	1.82(0.9360	0.210	1.84(0.931)	0.070	1.79(0.955)	
	Control	1.41(1.103)		1.62(1.18)		1.41(1.13)		1.48(1.14)	
Habitual sleep efficiency	Case	2.82(0.673)	0.166	2.76(0.761)	0.247	2.76(0.799)	0.365	2.78(0.742)	
	Control	2.65(0.913)		2.61(0.968)		2.64(0.936)		2.63(0.936)	
Sleep disturbances	Case	1.24(0.605)	0.733	1.22(0.647)	0.762	1.22(0.623)	0.707	1.22(0.622)	
	Control	1.27(0.662)		1.25(0.656)		1.26(0.713)		1.26(0.675)	
Sleep medication	Case	0.45(0.926)	0.475	0.40(0.871)	0.393	0.42(0.890)	0.618	0.42(0.891)	
	Control	0.35(0.785)		0.30(0.711)		0.35(0.773)		0.33(0.755)	
Daytimedysfunction	Case	1.06(0.903)	0.046	0.96(0.912)	0.014	0.99(0.913)	0.098	1.01(0.905)	
	Control	1.34(0.88)		1.3(0.88)		1.23(0.95)		1.28(0.905)	

## Discussion

The impacts of the extremely low frequency (ELF) on sleep quality in high voltage substations in Kerman province were surveyed in a case–control study. The results indicated that sleep quality was poor in both groups, but larger number of case group had poor sleep quality than control group. This can be related to exposure to ELF electromagnetic fields. However, poor sleep quality was not significantly different between the two groups. The control group has less education level than the case group and this is why they are employed in difficult jobs. Hard work, poor working conditions, high workload, occupational status and fatigue are the causes of their poor sleep quality [34,35] .Electric and Magnetic field levels in the inspected substations were much below the standard levels for occupational exposure set by ICNIRP. This result is consistent with the findings of Korpinen study. They reported that occupational exposure to magnetic and electrical fields in different tasks in 110 KV high voltage substations has not gone beyond ICNIRP levels [36]. According to the present study, occupational exposure to average electric field intensity and magnetic flux density and PSQI total score revealed a direct relationship. This relationship can be also a reason for the poor sleep quality in the case group, and indicate that long-term exposure to ELF electromagnetic fields, even less than the permissible exposure limit, can lead to poor sleep quality. Our finding matches up with Sharifi’s study in which it was reported a long time exposure to magnetic fields in the 230 kV substations can cause to increase psychiatric disorders including sleep disorder, despite magnetic field levels were much below the reference levels of ICNIRP [9]. [8] reported the sleep disorders were directly associated with the magnetic field exposure. This study confirms there is a linear relationship between exposure to magnetic fields and sleep disorders [8].

Average time to fall asleep and hours of actual sleep for case group were more than the control group. Electrical substations operators spent more time in bed without sleeping than others. Our finding can approve the effect of ELF electromagnetic fields on sleep quality. Akerstedt in 1999 assessed the sleep parameters using an electroencephalogram (EEG). They showed continuous exposure of healthy subjects to 50 Hz frequency and0.1 mG magnetic fields at the night resulted in sleep disorders. Total sleep time; sleep efficiency, sleep slow waves (stages III and V) and slow-wave activity decreased significantly related to magnetic field exposure [34]. In 1999, Graham and Cook also implied that the intermittent exposure to 60 Hz frequency and 2.8 mG magnetic fields can result in decreasing totalsleep time, decreasing sleep efficiency, increasing time in the second stage of sleep, reducing rapid eye movement (REM), and lengthening the delay to rapid eye movement (R.E.M.) sleep [37].

Although two above studies prove that magnetic field exposure severe reduction of total sleep time, but it is difficult to compare directly the two studies to ours because they examined the sleep quality in the laboratory by electro-encephalogram and under controlled conditions, and also there was0.1 mG continuous exposure [34] and 2.8 mG intermittent exposure [8] to magnetic fields, whereas the sleep quality of the present study was evaluated by questionnaire and there was continuous exposure to magnetic flux density 2.32, 7.49 and 7.51 mG.

Seung Cheol Hong reported that there is no significant difference between the exposure to ELF electromagnetic fields and the mean sleep duration and waking times. The magnetic field levels, on average, were 0.7 μT in head, 8.3 μT in lumbar and 3.5 μT in leg. An electrical page produced the magnetic fields and 9 people were studied for 11 weeks [38]. The various work conditions, method for assessing sleep quality, devices which produce electromagnetic radiation, the number of individuals, magnetic flux density and duration of exposure can be the reasons for difference between the mentioned study and our findings.

Roosli in 2004 reported that among symptoms attributed to electromagnetic field exposure, sleep disorders (58%), headaches (41%), nervousness or distress (19%), fatigue (18%), and concentration difficulties (16%) were the most common complaints [39] .

Schreier showed a 5% prevalence of hypersensitivity to electromagnetic fields. The most complaints were related to sleep disorders (43%) and headache (34%) [40]. Wagner observed no statistically significant difference in sleep quality within participants who were or not were exposed to electromagnetic fields. Radio frequency (RF) (900 MHz and 217 KHz) was used and there was the continuous exposure to them at night. Electro-encephalogram was applied to evaluate the sleep quality [41]. Hutter assessed subjective symptoms, sleeping problems, and cognitive performance in subjects living near mobile phone base stations. Total HF-EMF and exposure related to mobile telecommunication were far below recommended levels (max. 4.1 mW/m2). Average power density was slightly higher in the rural area (0.05 mW/m^2^) than in the urban area (0.02 mW/m^2^). Despite the influence of confounding variables, including fear of adverse effects from exposure to HF-EMF from the base station, there was a significant relation of some symptoms to measured power density; this was highest for headaches [42].

## Conclusion

The results presented in this paper showed a positive correlation coefficient between occupational exposures to ELF electromagnetic fields and sleep quality score, so it cannot reject the impact of the fields on sleep quality. Since other finding indicated the weak electromagnetic fields can have the biological effects and reducing work hours can prevent their biological effects.

Finally, exposure to electromagnetic fields or EMF has raised concern on the possible health effects of them; therefore they have become an interest issue for a great numbered people (especially people who live near power lines) and are an active area of biophysical research; but for providing more accurate results, much more research is needed.

Although many factors can affect sleep quality, it was tried to eliminate the maximum of confounding variables. It is suggested to complete this study in subsequent research, for example it is useful to measure the impact of job stress variables (such as: overtime, low social support, physically grueling jobs, part-time work) on sleep quality [43-45]. In this study, subjective sleep quality was measured, but for more accurate results it is necessary to assess the objective factors of sleep in high voltage substations using polysomnography that is a gold- standard test for the diagnosis of sleep disorders.

## Nomenclature

PSQI: Pittsburgh sleep quality index; EMF: Electric and magnetic field; ELF: Extremely low-frequency; ELFMF: Extremely low-frequency magnetic field; ICNIRP: International commission on non-ionizing radiation protection.

## Competing interests

The authors declare that they have no competing interests.

## Authors’ contributions

MRM: conception and design, analysis and interpretation, administrative, technical, or material support, supervision. TB: conception and design, data collection, analysis and interpretation, writing the manuscript. AAH: conception and design, analysis and interpretation, statistical expertise. MRG: conception and design, critical revision of the manuscript, administrative, technical, or material support. SFD: data collection, administrative, technical, or material support, writing the manuscript.
